# Carriage of ESBL-producing Gram-negative bacteria by flies captured in a hospital and its suburban surroundings in Ethiopia

**DOI:** 10.1186/s13756-020-00836-0

**Published:** 2020-11-04

**Authors:** Tafese Beyene Tufa, Andre Fuchs, Tobias Wienemann, Yannik Eggers, Sileshi Abdissa, Marlen Schneider, Björn-Erik Ole Jensen, Johannes G. Bode, Klaus Pfeffer, Dieter Häussinger, Colin R. Mackenzie, Hans Martin Orth, Torsten Feldt

**Affiliations:** 1 College of Health Sciences, Arsi University, P.O. Box 04, Asella, Ethiopia; 2 Hirsch Institute of Tropical Medicine, P.O. Box 04, Asella, Ethiopia; 3 Department of Gastroenterology, Hepatology and Infectious Diseases, Düsseldorf University Hospital Center, Moorenstr. 5, 40225 Düsseldorf, Germany; 4 Institute of Medical Microbiology and Hospital Hygiene, Düsseldorf University Hospital Centre, Universitätsstr. 1, 40225 Düsseldorf, Germany; 5 Department of Infectious Diseases, Tropical Medicine, Nephrology and Rheumatology, St. Georg Hospital, Delitzscher Str. 141, 04129 Leipzig, Germany

**Keywords:** Flies, Colonization, Multidrug resistance, Transmission, One health, Africa, Antimicrobial resistance

## Abstract

**Background:**

Local data from the Asella Teaching and Referral Hospital in the town of Asella, Ethiopia reveal a high prevalence of extended-spectrum β-lactamase- (ESBL) producing Gram-negative bacteria (GNB) in clinical isolates. To investigate a possible route of transmission, we determined the proportions ESBL-producing GNB in isolates from flies caught in the hospital and in the town of Asella.

**Methods:**

Flies were collected in August 2019 from the neonatal intensive care unit (NICU), the orthopedic ward, the hospital’s waste disposal area, and from a butchery situated 1.5 km from the hospital. After trapping, the flies were macerated and suspended in sterile normal saline. The suspensions were inoculated on MacConkey agar and incubated overnight. Species identification and antimicrobial susceptibility testing were performed using Vitek®-MS and VITEK® 2.

**Results:**

In total, 103 bacterial isolates were obtained from 85 flies (NICU: 11 isolates from 20 flies, orthopedic ward: 10 isolates from 12 flies, waste disposal area: 37 isolates from 26 flies, butchery: 45 isolates from 27 flies). The proportions of ESBL-producing bacteria among isolates obtained from flies collected in the hospital compound were significantly higher (82%, 90%, and 57% in NICU, orthopedic ward and waste disposal area, respectively) compared to flies collected outside of the hospital compound (2% (@1/45) in the butchery) (*p* ≤ 0.001). The proportion of ESBL was 67% (6/9) among *Raoultella* spp*.* 67% (4/6) among *Kluyvera* spp*.,* 56% (5/9) among *Enterobacter* spp., 50% (5/10) among *E. coli*, and 44% (8/18) among *Klebsiella* spp*.*. Of the 40 ESBL-genes detected, 85% were CTX-M-like, 83% TEM-like, 23% SHV-like, and 2% CTX-M-2-like. ESBL-producing bacteria showed higher rates of resistance against ciprofloxacin (66% vs. 5%), gentamicin (68% vs. 3%), piperacillin-tazobactam (78% vs. 5%), and trimethoprim-sulfamethoxazole (88% vs. 16%), compared to non-ESBL-producing bacteria.

**Conclusion:**

A high proportion of ESBL was identified in isolates from flies caught in the hospital compound compared with isolates of flies collected at a distance of 1.5 km from the hospital. Flies can be potential vectors for transmission of multidrug-resistant (MDR) bacteria within hospitals. Further studies are needed to determine the source of MDR colonization in flies and possible impact of MDR for nosocomial infections.

## Introduction

The flies have been proposed to be a potential vector for communicable diseases and multidrug resistance (MDR) in hospitals, particularly in developing countries [1, 2]. Flies can transmit MDR microorganisms in the three ways: mechanical translocation, regurgitation (bio-enhanced transmission) and defecation, in which MDR bacteria may become a part of the gut flora of flies thus carrying bacteria for the life span of the fly and contaminating their environment via feces and/or regurgitation. If flies play a role in AMR transmission, current hospital hygiene programs, focusing on patient isolation, hand hygiene, and antimicrobial stewardship programs, may not be sufficient to address the expansion of antimicrobial resistance (AMR), especially in resource limited, and possibly fly-abundant, settings [1, 3].

The expansion of MDR due to extended spectrum β-lactamase- (ESBL) production in Gram-negative bacteria (GNB) has become an emerging threat to antibiotic treatment success in resource limited settings. ESBLs are enzymes, encoded by genes often found on mobile genetic elements, which mainly include class A β-lactamases, such as CTX-M-type, TEM, and SHV and they confer resistance to the penicillin and cephalosporin antibiotic classes. CTX-M-type β-lactamases are the most abundantly found ESBL enzymes worldwide [4].

Ceftriaxone and ceftazidime are the most commonly used antibiotic substances for the treatment of blood stream infections caused by GNB at the study site. Local data from the Asella Teaching and Referral Hospital (ATRH) reveal a high prevalence of ESBL-producing GNB in clinical isolates, hampering the efficacy of empiric antibiotic therapy [5]. To our knowledge, there are no reports on the colonization rate of flies with ESBL-producing bacteria in Ethiopian hospitals and the possible implications for the spread of AMR. A prospective study was therefore initiated to investigate the colonization of flies with ESBL-producing GNB at the ATRH compound and in Asella town.

## Methods and materials

The flies were collected in August 2019 in the ATRH’s neonatal intensive care unit (NICU), the orthopedic ward, the hospital’s waste disposal area, and in a butchery located 1.5 km from the hospital. The flies were trapped with non-toxic retail flycatchers (Profissimo® Giftfreier Fliegenfänger, Germany) and stored in 2 ml of sterile normal saline within the same day.

For further analysis in this study, only animals displaying essential taxonomic morphologic criteria of flies such as size, shape and color were selected.

After maceration in sterile saline the suspensions were inoculated on MacConkey agar and incubated at 37 °C for 18–24 h. All colonies growing on MacConkey agar were isolated and sub-cultured for species identification. All phenotypically different colonies obtained from one fly were subcultured. The isolates were preserved at − 81 °C in the Microbank® vials (Pro-Lab Diagnostics Inc., Toronto, Canada) and transported to Germany for identification using MALDI-ToF-MS (VITEK®-MS, bioMérieux, Marcy-l’Étoile, France) and antimicrobial susceptibility testing (AST) with VITEK® 2 (bioMérieux) and Kirby-Bauer for confirmation of some results by VITEK 2 (performed at the Institute of Medical Microbiology and Hospital Hygiene, Heinrich Heine University Düsseldorf, Germany). All results were interpreted according to European Committee on Antimicrobial Susceptibility Testing (EUCAST) version 2018 V8.0. All bacteria positive in the VITEK ESBL phenotype screening were subjected to molecular detection using PCR as detailed below.

After identification, the bacterial DNA was prepared by producing a suspension of a pure colony from MacConkey agar in 200 μL of Tris–EDTA pH 7.5. The suspension was then heated at 95 °C for 10 min, followed by centrifugation at 10,000 rpm for 2 min. Then 150 µl the supernatant was transferred to the new 1.5 ml tube and was stored at − 20 °C until PCR testing.

Identification of bacterial resistance genes was performed by PCR of ESBL-gene sequences common to groups of ESBL types. Bacterial strains with suspected production of ESBL were investigated by PCR, following the protocols described by Strauß et al. for identification of the β-lactamase (*bla*) CTX-M, *bla*_SHV_ and *bla*_TEM_ genes [6].

The frequency of ESBL genes detected in isolates from flies’ colonization was compared with the frequency of ESBL genes previously detected in clinical isolates from patients with acute infectious diseases or sepsis from the same hospital (unpublished data). *Escherichia coli* and *Klebsiella pneumonia* were the most common GNB isolated from blood, urine and wound swabs which were used as the clinical isolates to compare the proportion of ESBL frequency with GNB colonized flies. The same method was followed for identification and AST result interpretation for the bacteria isolated from clinical samples and flies.

IBM SPSS Statistics for Windows, version 25 (IBM Corp., Armonk, N.Y., USA) was used for statistical analysis.

## Results

A total of 103 bacterial isolates were obtained from 85 flies (NICU: 11 isolates in 20 flies, orthopedic ward: 10 isolates in 12 flies, waste disposal area: 37 isolates in 26 flies, butchery: 45 isolates in 27 flies). *Klebsiella* spp., and *Proteus* spp. were among common pathogenic bacteria isolated in the butchery. However, nearly half of the bacteria isolated from flies caught at the butchery in Asella town were not commonly pathogenic for humans. The frequency of ESBL-production among isolated bacteria from flies caught at the different study sites was variable (see Table 1).

**Table 1:** Frequency of ESBL-producing Gram-negative bacteria in 85 flies caught in at ATRH and Asella town, Ethiopia

Site	Isolates (n)	Rate of ESBL- n (%)
NICU	11	9 (82%)
Orthopedic ward	10	9 (90%)
Waste disposal area	37	21 (57%)
Butchery	45	1^a^ (2%)

^a^We found only a single *Escherichia coli* ESBL producing from the butchery

**Fig. 1:**
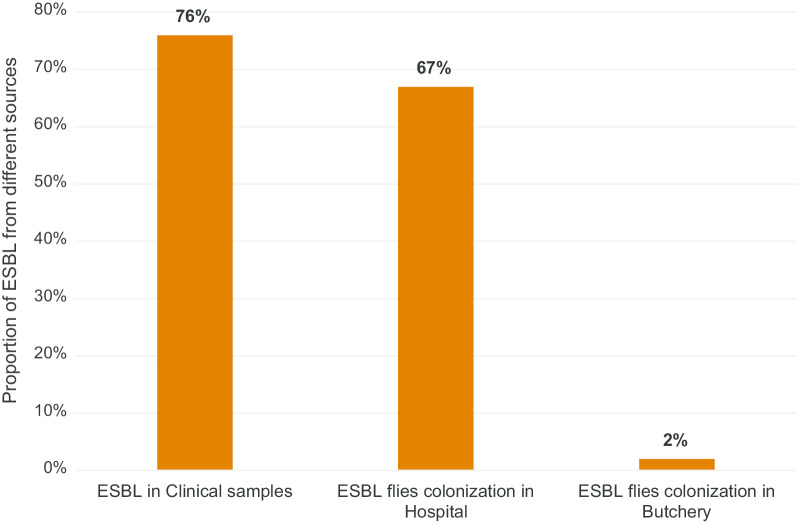
Comparison of ESBL frequency in clinical samples and flies colonization. Source of clinical samples data: ESBL in clinical isolates of Gram-negative bacilli at Asella teaching and referral hospital, central Ethiopia (2016 to 2019). The proportion of ESBL flies colonization in hospital was significantly different compared with ESBL flies colonization in butchery (67% vs. 2%; *P* value < 0.001)

The proportion of ESBL producing bacteria among GNB isolates from flies was 9 (82%), 9 (90%), and 21 (57%) in NICU, orthopedic ward and waste disposal area in the hospital’s compound, respectively. Only one isolate with ESBL-carriage was identified in flies from the butchery. Overall, the proportion of ESBL-producing bacteria colonized flies in the hospital compound was 39 (67%). The different colonization rates with ESBL of flies trapped inside and outside the hospital compound was statistically significant (*p* ≤ 0.001) (see Fig. 1).

The proportion of ESBL expression was 67% (6/9) in *Raoultella* spp*.*, and 67% (4/6) in *Kluyvera* spp*.,* 56% (5/9) in *Enterobacter* spp., 50% (5/10) in *E. coli and Citrobacter* spp*.* and 44% (8/18) in *Klebsiella* spp*.*, respectively (Table 2).

**Table 2:** Proportion of ESBL expression among GNB isolates from flies trapped from hospital compound and butchery

Bacterial species	ESBL positive n (%)	ESBL negative n (%)	Total n
*Raoultella* spp.	6 (67%)	3 (33%)	9
*Kluyvera* spp.	4 (67%)	2 (33%)	6
*Enterobacter* spp.	5 (56%)	4 (44%)	9
*Escherichia coli*	5 (50%)	5 (50%)	10
*Citrobacter* spp.	5 (50%)	5 (50%)	10
*Klebsiella* spp.	8 (44%)	10 (56%)	18
*Providencia* spp.	2 (29%)	5 (71%)	7
*Proteus* spp.	1 (13%)	7 (87%)	8
*Moellerella wisconsensis*	1 (10%)	9 (90%)	10
Others^a^	3 (30%)	7 (70%)	10

^a^Others (one isolate each): *Comamonas testosteroni, Pantoea agglomerans,* and *Rahnella aquatilis* (ESBL expression); *Aeromonas hydrophila, Cedecea davisae, Hafnia alvei, Leclercia adecarboxylata, Lelliottia amnigena, Serratia liquefaciens,* and *Yokenella regensburgei* (no ESBL expression); Even though we found four isolates of *Acinetobacter* spp. from flies, the proportion of ESBL was not analyzed in this study. As described in (Fig. 1) above, the proportion of ESBL was near to similar in GNB isolated from clinical samples and flies caught in hospital compound. However, it was extremely low 1 (2%) in flies caught in butchery

Among bacteria carrying ESBL isolated from flies in this investigation, 85% (n = 34) carried CTX-M-like, 83% TEM-like (n = 33), 23% SHV-like (n = 9) and 1 CTX-M-2-like genes. CTX-M-9- and CTX-M-8/25-like genes were not detected. The comparison of the detection frequency of the different major ESBL genes of GNB isolated from blood, urine and wound swab samples between 2016 and 2019 from the same hospital (own data, not published) and of bacterial isolates from flies shows clear similarities (Table 3). However, CTX-M-9 was only detected from clinical specimens and CTX-M-2 was only detected from isolates from flies.

**Table 3:** Comparison of frequency and characterization of ESBL genes from clinical isolates (n = 32) and isolates from flies (n = 40)

	ESBL genes					
	Total	CTX-M-1 n (%)	TEM n (%)	SHV n (%)	CTX-M-9 n (%)	CTX-M-2 n (%)
ESBL in clinical isolates	32	26 (81%)	22 (69%)	7 (22%)	2 (6%)	0
ESBL in isolates from flies	40	34 (85%)	33 (83%)	9 (23%)	0	1 (3%)

ESBL, extended spectrum β-lactamases; CTX-M, cefotaximase-Munich; SHV, sulfhdryl variable; TEM, Temoniera

**Fig. 2:**
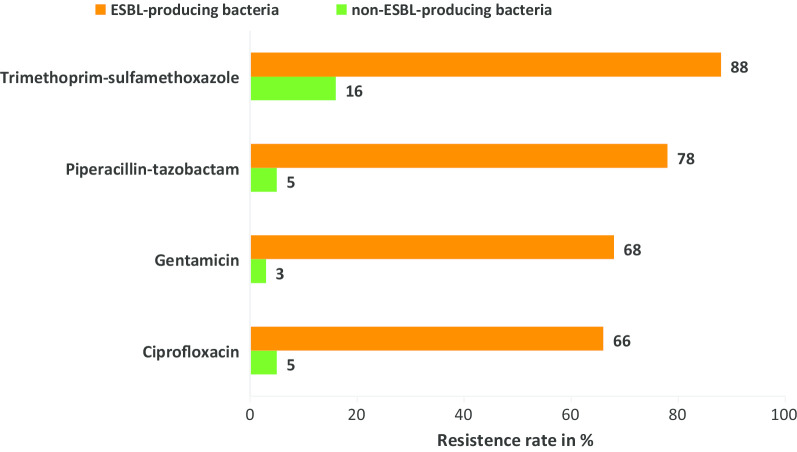
Resistance rate among ESBL- (n = 40) and non-ESBL-producing (n = 62) Gram-negative bacteria isolated from flies

Among isolated bacteria, phenotypical AMR against non- β-lactam antibiotics used for treatment of other ESBL-producing bacteria was very high. ESBL-producing bacteria showed a higher rates of AMR against ciprofloxacin (66% vs. 5%, *p* < 0.001), gentamicin (68% vs. 3%, *p* < 0.001), piperacillin-tazobactam (78% vs. 5%, *p* < 0.001), and trimethoprim-sulfamethoxazole (88% vs. 16%, *p* < 0.001) compared to non ESBL-producing bacteria (Fig. 2).

## Discussion

Recently, flies were recognized as potential vectors for AMR in hospital and non-hospital environments [7, 8]. In the study center, the proportion of ESBL-producing GNB isolated from clinical samples was very high (Fig. 1). We conducted this study in order to compare the colonization of flies with ESBL-producing GNB at various locations inside and one location with high density of flies outside of the hospital compound. We found a high proportion of ESBL-producing bacteria among isolates from flies collected inside the hospital compared to the near absence of ESBL genes in bacterial isolates from flies collected 1.5 km away from the hospital. The ESBL proportion was highest at the NICU and at the orthopedic ward, and only slightly lower at the hospital waste disposal area (Table 1). Our findings could partly be explained by exposition of bacteria to different antibiotics in the environment of the hospital or more likely by the accumulation of resistant bacteria in and on flies in the patients’ environment. Similar to our findings, a study conducted in Iran shows that bacterial isolates from houseflies in a hospital compound had a significantly higher frequencies of antimicrobial resistance against various antibiotics, than bacteria isolated from houseflies in non-hospital environment [7]. A study conducted in Berlin, Germany showed that the prevalence of ESBL in flies trapped from two different residential areas differed (0% vs. 18%) [9]. According to this study, the distribution of ESBL-producing bacteria among flies in certain geographical locations is not uniform.

In our study, the proportion of ESBL-producing GNB was very high among common pathogenic bacteria like *Escherichia coli, Klebsiella* spp., *Enterobacter* spp., *Citrobacter* spp., and *Raoultella* spp. compared with opportunistic bacteria. *Kluyvera* spp*.* are opportunistic bacteria with the highest rate of ESBL-production (Table 2). Half of the bacteria isolated from the butchery in Asella town were not commonly pathogenic bacteria. This different distribution of bacteria colonizing the isolated flies might also influence the proportion of ESBL isolates from different sites based on pathogenicity of the bacteria and their exposure to cephalosporin antibiotics [10].

In this study, the most frequently detected resistance genes in confirmed ESBL-producing GNB colonized flies were CTX-M-1-like gene, followed by TEM-like gene and SHV-like gene, respectively. The frequency and characterization of ESBL genes of clinical samples and flies isolates showed similarities (Table 3) [11] and also similar findings reported by Boulesteix et al. [12] from Dakar, Senegal. This suggests that flies may acquire the bacteria from the hospital environment. Similar findings were reported by Fotedar et al. [13]; however, to clearly identify the source of the ESBL-producing bacteria on flies needs further investigation. On the basis of our results, no statement can be made on the question of whether flies can be considered as vectors for MDR bacteria. In this context, however, it is interesting to note that Rahuma et al. [14] reported earlier that flies may be potential vectors for the transmission of MDR bacteria from hospitals to surrounding communities. This may suggest that flies colonized with ESBL-producing bacteria found in the hospital compound can possibly spread AMR to surrounding residential areas or restaurants, thereby endangering public health [15]. As described in Table 2, not only well-known pathogenic bacteria but also opportunistic bacteria can carry clinically relevant resistance genes and enhance the spread of AMR in the community.

Published investigations from Ethiopia demonstrate that MDR GNB commonly express the *bla*_CTX-M-1_ gene encoded in ESBLs and the *bla*_NDM-1_ gene in carbapenemase-producing bacteria [5, 16–18]. For molecular detection of ESBL, *bla*_CTX-M-1_ ESBL gene can be used as target gene by either conventional PCR or a loop-mediated isothermal amplification (LAMP) technique, which is rapid, effective and affordable to detect the presence of *bla*_CTX-M-1_ ESBL gene in RLS like Ethiopia [19].

In this study, the susceptibility to non-β-lactam antibiotics such as ciprofloxacin, gentamicin, and trimethoprim-sulfamethoxazole was significantly lower in ESBL-producing bacteria compared to ESBL-negative bacteria (*p* < 0.001). This finding is an indicator for the limited options of appropriate antibiotic therapy regimen for ESBL-producing bacterial infection management. Poudel et al. [20] also reported high rates of resistance of *E. coli* and *K. pneumoniae* isolated from flies against tetracycline and ampicillin due to emergence of ESBL-producing strains. This might probably be caused by plasmid-mediated mobile resistance genes such as quinolone-resistance *(qn*r) genes, aminoglycoside acetyltransferase (*aac*), *dfr* (trimethoprim resistance) and *sul* (sulfamethoxazole resistance) genes, being more frequent in ESBL-producing bacteria compared to ESBL-negative bacteria [21–24]. However, the identification of other resistance genes than the described ESBL genes was not part of this investigation.

In tropical regions where poor hospital hygiene is common, hand hygiene and patient isolation or implementation of antimicrobial stewardship programs may not be sufficient to control the expansion of AMR. Our findings can be considered as indicators for a possible dissemination of antimicrobial resistance inside and outside of hospital compounds and to the nearest environments by flies. Therefore, to tackle the expansion of ESBL-producing bacteria, fly-control measures in critical areas of the hospitals might be essential [7, 15]. In order to inhibit further expansions of ESBL-carrying bacteria from hospitals to residential areas, environmental and health professionals and municipality administration should work together and strengthen a one health approach. Future AMR prevention and control protocols may consider screening of flies for AMR and eradication-measures to control the population density of flies at health care facilities in tropical regions [20]. As distribution of ESBL genes in clinical samples and flies caught in the hospital show comparable results, flies might be used as an indicator organism for ESBL-prevalence in hospital facilities.

Our study has certain limitations. Fly species identification was not performed and we recognize that the different species *Musca sorbens* and *Musca domestica* have a different life style and thus may be involved in bacterial transmission to different degrees. Nevertheless, an identification of the different species in this study is unlikely to have an impact on the results in general. ESBL-colonization in the community was reported as low, but flies were sampled from a single butchery only. Even though the colonization with ESBL to be common in flies at the hospital, the source of the ESBL was not addressed. The study design also fails to point out whether the external organs like legs and mouth or the gut of the flies are more involved in carrying ESBL-producing bacteria, a factor with possible impact for the transmission of the bacteria. The role of flies in transmission of nosocomial infections and the source of ESBL-producing bacteria in the hospital needs further investigation.

A further limitation is the use of non-selective media for the screening process in flies and the fact that we tested phenotypically different isolates obtained from one fly (in some case we found more than one bacteria from a single fly). This procedure was chosen in order to harmonize the study protocol with diagnostics in the clinical setting. In consequence, our data reflect the proportion of ESBL among GNB isolates from flies, but not the prevalence of ESBL-carriage among flies, which might have been higher, if a selective screening approach was used. However, even with the non-selective culture technique used, ESBL-producing bacteria were detected in a major proportion of flies.

## Conclusions and recommendations

A high proportion of flies trapped within the hospital compound were colonized with ESBL-producing bacteria, whereas ESBL-production was nearly absent among flies collected in a butchery 1.5 km away from the hospital. The flies may be a relevant factor in the spread of MDR microbes in hospitals or hospitals surroundings. Antibiotic susceptibility to ciprofloxacin, gentamicin, and trimethoprim-sulfamethoxazole was lower in ESBL-producing bacteria compared with ESBL-negative bacteria which can limit treatment options. Antimicrobial resistance prevention and control protocols should consider the role of flies in hospitals in tropical regions. Our findings warrant the need of a one health approach to minimize the spreading of MDR strains to the environment. Further studies are needed to determine the role of flies as vectors for MDR nosocomial infections.

## Data Availability

All data generated or analyzed during this study are included in this published article.

## Abbreviations

aac: Aminoglycoside acetyltransferase
AMR: Antimicrobial resistance
AST: Antimicrobial susceptibility testing
ATRH: Asella Teaching and Referral Hospital
CTX-M: Cefotaximase-Munich
dfr: Dihydrofolate reductases
EDTA: Ethylene diamine tetraacetic acid
ESBL: Extended spectrum β-lactamase
EUCAST: European Committee on Antimicrobial Susceptibility Testing
GNB: Gram-negative bacteria
LAMP: Loop-mediated isothermal amplification
MALDI-TOF: Matrix-assisted laser desorption/ionization time-of-flight mass spectrometry
MDR: Multidrug-resistant
NDM: New Delhi metallo-β-lactamase
NICU: Neonatal intensive care unit
PCR: Polymerase chain reaction
qnr: Quinolone-resistance
RLS: Resource limited settings
sul: Sulfamethoxazole resistance gene
SHV: Sulfhydryl variable
TEM: Temoniera
